# Influence of the Chloride Attack on the Post-Cracking Behavior of Recycled Steel Fiber Reinforced Concrete

**DOI:** 10.3390/ma14051279

**Published:** 2021-03-08

**Authors:** Cristina Frazão, Joaquim Barros, José Alexandre Bogas

**Affiliations:** 1 Institute for Sustainability and Innovation in Structural Engineering (ISISE), Department of Civil Engineering, School of Engineering, University of Minho, 4800-058 Guimarães, Portugal; barros@civil.uminho.pt; 2 Civil Engineering Research and Innovation for Sustainability (CERIS), Department of Civil Engineering, Architecture and Georesources, Instituto Superior Técnico, University of Lisbon, 1049-001 Lisbon, Portugal; abogas@civil.ist.utl.pt

**Keywords:** RSFRC, recycled steel fibers, chloride-induced corrosion, post-cracking behavior, constitutive laws

## Abstract

The main purpose of the present work is to study the mechanical behavior and durability performance of recycled steel fiber reinforced concrete (RSFRC) under a chloride environment. To this end, the effect of chloride attack on the load-carrying capacity of pre-cracked RSFRC round panels is investigated by performing round panel tests supported on three points (RPT-3ps), considering the influence of the crack width and the fiber distribution/orientation profile. In addition, the influence of the adopted chloride exposure conditions on the post-cracking constitutive laws of the developed RSFRC is also assessed by performing numerical simulations for the prediction of the long-term performance of RSFRC under these aggressive conditions. The tensile stress–crack width relationship of RSFRC is derived by performing an inverse analysis with the RPT-3ps results. The obtained experimental and numerical results show a negligible effect of the chloride attack on the post-cracking behavior of RSFRC for the chloride exposure conditions and pre-crack width levels adopted in this study.

## 1. Introduction

In recent years, several investigations have explored the potential of end-of-life tires by-products in the construction industry, such as the use of recycled steel fibers (RSFs) in the reinforcement of cement-based materials [1,2,3,4,5,6,7], namely, in fiber reinforced concrete (FRC). FRC is being used in slabs and shells, such as the case of flooring and tunneling since the support redundancy of this type of structure favors the occurrence of a high level of stress redistribution during crack propagation, which increases their ultimate load regarding their cracking load [8,9]. These potentialities are being considered for using FRC in offshore applications [10,11].

According to the literature, RSFs show a high potential to be an effective concrete reinforcement for application in structural elements, namely, those that are exposed to coastal/marine environments [1,2,3,4,5,6,7]. However, research on the corrosion resistance of recycled steel fiber reinforced concrete (RSFRC) is almost non-existent, namely, concerning the effects of chloride attack on the fiber reinforcement mechanisms developed during the fiber pull-out from the matrix in cracked RSFRC.

Chloride attack is one of the main deterioration mechanisms of reinforced concrete, especially in countries with a large coastline (involving offshore and onshore constructions in the marine environment). Under this harsh environment, the service life of reinforced concrete is essentially governed by the depassivation and subsequent corrosion of steel reinforcement. In this context, steel fiber reinforced concrete under this action must be assessed, namely, considering the low fiber concrete cover and cracking effects, which may expose and impair the fiber properties.

For conventional steel fiber reinforced concrete (SFRC), literature in chloride-induced corrosion resistance is mainly focused on corrosion arising from the cracking process [12,13,14,15]. According to Marcos-Meson et al. [15], four stages may be identified on the impact of load-induced cracks on the damage of the fiber-matrix interfacial transition zone (ITZ) promoting fiber corrosion, which are (1) in uncracked SFRC, the steel-matrix ITZ acts as a protective coating of steel fibers surface, preventing the access of aggressive agents; (2) the matrix cracks when its tensile capacity is attained, which activates the fiber–matrix bond with detrimental consequences on the fiber–matrix ITZ performance, providing a preferential path for transport of chlorides, metal ions, and oxygen that promotes corrosion mainly at the fiber’s area crossing the crack; (3) if up to a critical crack width the fiber does not reach a critical slipping, the damage at the fiber–matrix interface would eventually heal [16], and the expansion of the corrosion products increases the fiber roughness, which may improve the fiber–matrix frictional bond [17] and hence the residual tensile capacity of the corresponding SFRC [12]; and (4) larger fiber slipping leads to defective healing and excessive damage at the ITZ, conducting to a progressive and localized reduction of the fiber cross section due to corrosion. When the tensile capacity of the fiber cross section is lower than the fiber pullout strength, the fiber tensile rupture becomes the governing failure mode in the crack propagation of an SFRC element, with a decrease of its post-cracking load-carrying capacity and deformation performance [13]. This tensile rupture can be even anticipated in case the fiber is subjected locally to axial, shear, and bending at its exit point, which is a common situation of fibers inclined toward the crack that is crossing [18]. If the fiber is corroded, this premature rupture can be even anticipated.

In general, the addition of RSFs has a negligible effect on the diffusion of chloride ions into uncracked concrete, and the critical chloride content corresponding to the beginning of fiber corrosion tends to be higher than that found in conventional reinforced concrete structures [3]. According to Marcos-Meson et al. [15], the durability of cracked SFRC is controversially discussed in the literature; however, there is a general consensus regarding the high probability of corrosion on carbon-steel fibers bridging cracks wider than 0.5 mm, which leads to a significant reduction of the fiber cross section and causes notable reduction of the residual tensile strength due to a subsequent change in the failure mode from fiber pull-out to fiber failure, under moderate exposure to chlorides.

The RSFs’ reinforcement efficiency and the post-cracking behavior of RSFRC can be assessed by performing conventional material tests, including the double edge wedge splitting test [3,4,7] or the three-point notched beam bending test (3PNBBT) [5,6,7]. However, the stress–crack width (σ−ω) relationship, obtained from these tests is noticeably influenced by the number and orientation of fibers crossing the crack that propagates along the pre-notched plane [3]. Fiber orientation and distribution in FRC elements are significantly affected by the geometry of the element [19]. In prismatic elements, a larger number of fibers may be preferably oriented orthogonally to the fracture plane due to the wall effects caused by the geometry of the mold [8]. In this case, the fiber reinforcement is very effective, but it is only representative of a real FRC structure if the fiber distribution in the governing failure section of the structure can be represented by the one observed in the three-point notched beam bending test. FRC beams of relatively small cross-section width pertain to this class of structures [20]. In the case of slabs or shells, the fiber distribution is almost orthotropic, with the tendency of the fibers to be parallel to the middle plane of this type of structure, but in the plane, the fiber orientation is random [21]. For capturing the influence of the fiber orientation in this type of structures, a round panel test supported on three points (RPT-3ps) is the most recommended since three main cracks of different orientation are formed, which are crossed by fibers of representative orientation in real failure scenario of a slab or shell. Therefore, the design methodology of an RSFRC slab based on constitutive models derived from results obtained in RSFRC round panel tests (RPT-3ps) can ensure reliable simulations regarding the fracture properties of the RSFRC of the slab.

In this study, an experimental program was carried out to evaluate the effects of chloride attack on the load-carrying capacity of RSFRC round panels by performing RPT-3ps. The influences of the crack width and the fiber distribution/orientation profile on the force–deflection and energy dissipation responses obtained in RPT-3ps were investigated. Additionally, compressive tests and 3PNBBT were carried out to characterize the mechanical properties of RSFRC, namely, the compressive strength, the elasticity modulus, and the flexural behavior. Furthermore, by using the results determined in the RPT-3ps, the σ−ω relationship of the RSFRC was derived by inverse analysis. For this purpose, numerical simulations of RPT-3ps were executed combining a moment–rotation approach with a numerical model that considers the kinematic conditions of RPT-3ps at the failure stage and the equilibrium equations [22].

## 2. Experimental Program

### 2.1. Materials and Mix Composition

**Figure 1:**
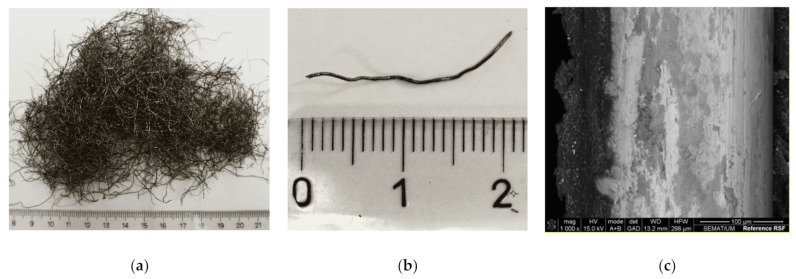
Recycled steel fibers (RSFs): (**a**) general view of multi RSFs; (**b**) general view of the geometry of a single RSF; and (**c**) SEM micrograph of the surface of a single reference RSF (magnification: 1000×) [3].

The recycled steel fibers (RSFs) used in this research were recovered by a shredding process of post-consumed truck tires. These RSFs generally have irregular shapes with various lengths and diameters (Figure 1a,b). The steel was separated from the rubber by an electromagnetic separator, and most of the RSFs still contain some rubber particles attached to their surface due to the shredding process (Figure 1c). According to a detailed characterization performed on a sample of 2000 fibers, on average, the RSFs have 20 mm in length ($l_{f}$), defined as the distance between the outer ends of the fiber, 0.25 mm in diameter ($d_{f}$), and an aspect ratio ($l_{f}/d_{f}$) of 110. The average tensile strength of RSFs, obtained from five fibers by means of direct tensile tests, was around 2648 MPa (coefficient of variation = 16%). A carbon concentration of 0.77% in the chemical composition of RSFs was determined by X-ray fluorescence spectrometry (XRF, X’Unique II spectrometer, Philips) analysis.

One RSFRC mixture was produced with CEM I 42.5R (C) according to EN 197-1:2011 [23], fly ash (FA), fine river sand (FS) (maximum aggregate size of 1.19 mm and fineness modulus of 1.91), coarse river sand (CS) (maximum aggregate size of 4.76 mm and fineness modulus of 3.84), crushed granite (CG) (maximum aggregate size of 19.1 mm and fineness modulus of 7.01), water (W), a polycarboxylate based superplasticizer (SP) with the commercial designation MasterGlenium SKY 617 (BASF Portuguesa, S.A., Prior-Velho, Portugal), and an RSF content of 1% in volume of concrete.

The mix design of RSFRC with the intended fresh and hardened properties was based on the packing density optimization method suggested in Barros et al. [24]. The composition of RSFRC is indicated in Table 1, for an RSFs content (Cf) of 75.8 kg/m^3^. To improve the sustainable character of the RSFRC, 40% of the volume of the binder was replaced by fly ash. The fly ash also improves the fresh stability and flowability of concrete due to the spherical shape of their constituent particles that act as micro-rollers, decreasing friction and flow resistance [25]. This mineral addition is also recognized to improve the long-term chloride penetration resistance of concrete [26].

### 2.2. Specimens Manufacture

According to ASTM C1550-08 [27], the nominal dimensions of the round panels are 800 mm in diameter and 75 mm in thickness. However, in order to facilitate handling and placing of the specimens, smaller RSFRC panels were produced with 600 mm in diameter and 60 mm in thickness. According to Minelli and Plizzari [28], this reduction of the panel’s diameter and thickness does not affect the scatter and repeatability of the test results. The round panels were cast with the RSFRC mixture detailed in Table 1. Two batches with the same composition (Table 1) were produced to cast 12 panels (six panels per casting). For each batch, four Φ150 mm × 300 mm cylindrical RSFRC specimens and three RSFRC beams with 600 × 150 × 150 mm^3^ were also cast for testing the relevant mechanical properties of the RSFRC. These specimens were water-cured until testing.

### 2.3. Test Procedures

#### 2.3.1. Mechanical Characterization of RSFRC

**Figure 2:**
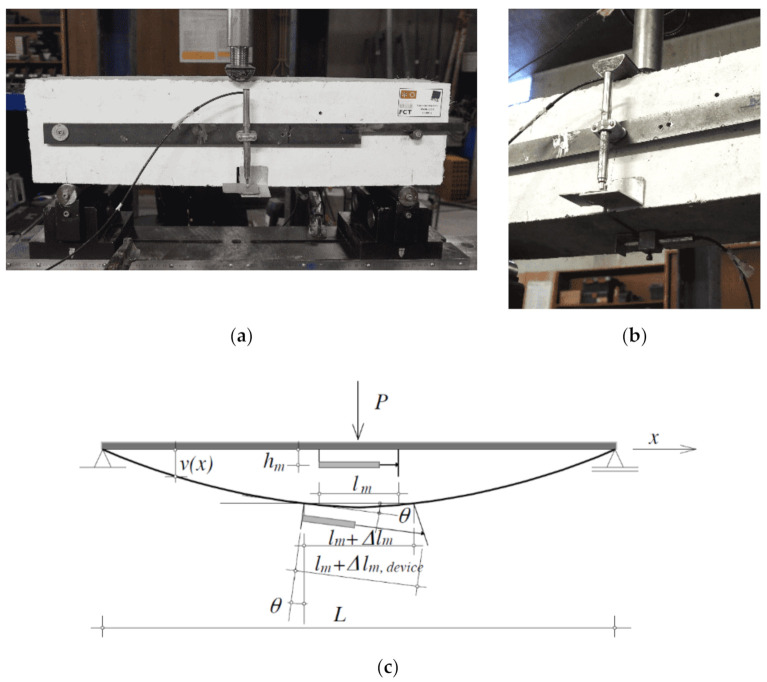
Test setup for three-point notched beam bending test (3PNBBT): (**a**) specimen front view; (**b**) specimen bottom view; and (**c**) measurement of non-linear tensile deformations—initial position of the measurement device and rotation of measurement points caused by rotation of the beam [33].

The compressive strength of the two batches of RSFRC, herein designated by “RSFRC_1 and RSFRC_2”, was assessed by testing the RSFRC cylindrical specimens under uniaxial compressive tests according to EN 12390-3:2011 [29]. Four specimens per batch were tested up to an axial strain level higher than the strain at peak stress in order to determine part of the strain-softening of the stress–strain ($\sigma_{c}-\varepsilon_{c}$) response of RSFRC. The modulus of elasticity was determined according to EN 12390-13:2014 [30] over three loading cycles, where the applied stress varied between 0.6 MPa and one-third of the estimated compressive strength. Axial deformations were measured by three linear variable displacement transducers (LVDTs with +/− 5 mm linear stroke), operating over an initial gauge length of 100 mm. The elasticity modulus and the stress–strain relationship were obtained in sets of four specimens tested at the same age of the round panel tests (120 days). The flexural behavior of RSFRC was assessed by testing three notched RSFRC beams with 600 × 150 × 150 mm^3^ (a notch depth of 25 mm, a span of 500 mm) per batch, under three-point loading conditions (3PNBBT) at the same age as the round panel tests (120 days). The method of casting the specimens and curing procedure, the position and dimensions of the notch, the load and specimen support conditions, the characteristics for both the equipment and measuring devices, and test procedure were those recommended by EN 14651 + A1 [31] and Model Code (MC) 2010 [32]. These tests were carried out under closed-loop displacement control at a constant rate of 3 µm/s, using the deflection measured at midspan as a control variable (Figure 2a). One additional LVDT was used to measure the crack mouth opening displacement (CMOD), which was placed on the bottom face of the beam at the mid-span (Figure 2b,c).

#### 2.3.2. Round Panel Tests

##### 2.3.2.1. Test Setup

**Figure 3:**
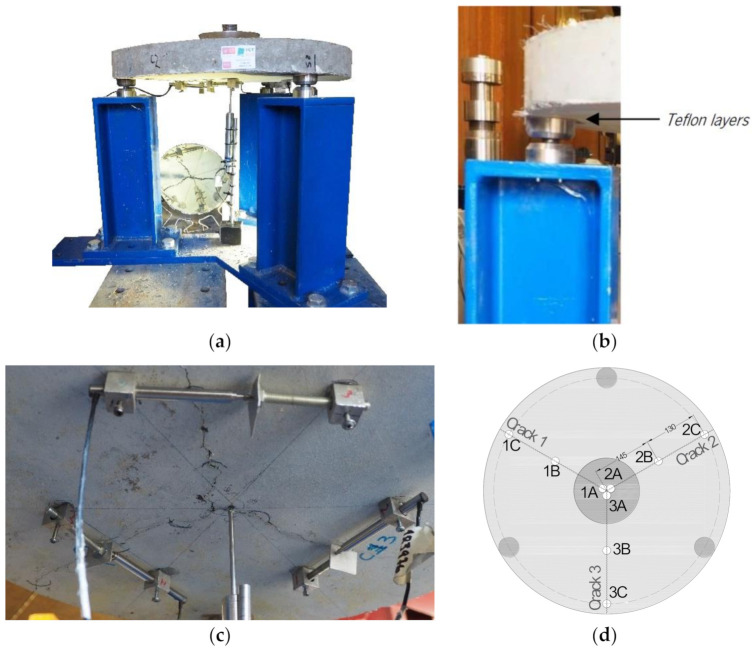
The round panel test supported on three points (RPT-3ps) setup: (**a**) the three pivots support system [3]; (**b**) connection between the panel and each pivot; (**c**) positions of the linear variable displacement transducers (LVDTs) for central deflection measurement and of LVDTs for crack width measurement; and (**d**) position of the crack width measurement with a USB microscope (“A”—At the center of the panel; “B”—At a distance of 145 mm from the panel center; “C”—At a distance of 275 mm from the panel center).

Round panel tests supported on three symmetrically arranged pivots (RPT-3ps) were conducted, as presented in Figure 3a. The connection between the panel and each pivot was provided by two round steel pieces of 50 mm diameter and 25 mm thickness, with a spherical seat of around 6 mm depth machined into the two surfaces to achieve the ball connection recommended by ASTM C1550-08 [27], as represented in Figure 3b. Two Teflon sheets were used between the concrete panel and each round steel plate to reduce friction (Figure 3b). The load was applied to the panel’s center through a hemispherical-ended steel piston at a constant displacement rate. The central deflection of the panel was measured by an LVDT installed at the bottom surface of the panel (Figure 3a). Three LVDTs were also used in the bottom face of the panel to measure the width of the three developed cracks (Figure 3c,d).

In the performed RPT–3ps, the influence of the crack width and the fiber distribution/orientation profile on the post-cracking behavior of RSFRC under chloride attack was investigated.

##### 2.3.2.2. Pre-Cracking Process

In order to investigate the influence of the crack width, the RPT-3ps was executed with pre-cracked RSFRC panels, for a target pre-crack width, $\omega_{cr}$, of about 0.5 mm and 1.0 mm. To this end, the following procedure was adopted: (1) impose a deflection rate of 1.0 mm/min up to reach a central displacement of 2.5 mm and unload the panel; (2) check if the $\omega_{cr}$, corresponding to the average value measured by the three LVDTs shown in Figure 3c, is close to the intended one; (3) if not, impose successive increments of 0.25 mm to the installed central displacement, at the same deflection rate, until the intended pre-crack width is achieved. At the end, and before submitting the panels to the environmental exposure, the $\omega_{cr}$ was measured with a USB microscope in the nine points indicated in Figure 3d (three points at each crack).

##### 2.3.2.3. Environmental Exposure

In order to study the influence of chloride attack in the post-cracking behavior of RSFRC, the pre-cracked panels were immersed in a 3.5 wt% NaCl (SALEXPOR, Olhão, Portugal) solution at a constant temperature of 20 °C. The period adopted for the chloride exposure was 90 days of dry-wet cycles, consisting of three days wetting and four days drying. For comparison purposes, pre-cracked reference panels were also immersed in tap water at a constant temperature of 20 °C for the same exposure period defined for chloride attack. According to Marcos-Meson et al. [15], the use of dry–wet cycles proved to be an effective method to accelerate corrosion-induced damage of SFRC.

The period of 90 days for chloride exposure was adopted based on a preliminary study carried out to characterize the corrosion resistance of RSFs caused by chloride attack. In this preliminary study, the mass loss of single RSF by corrosion was evaluated after dry–wet cycles of three days wetting and four days drying in a 3.5 wt% NaCl solution for 90 days. To simulate the exposure of RSFs bridging a crack of 0.5 mm and 1.0 mm width, fibers were painted with Lacomit varnish (Agar Scientific Ltd., Stansted, UK) except for a length of 0.5 mm and 1.0 mm, located at half-length of RSFs, in an attempt of restricting the corrosion to this unpainted zone. For comparison purposes, RSFs were also completely exposed to this chloride medium.

This was a simplified test since RSFs’ corrosion did not occur in a concrete environment. In this case, it was assumed that the concrete crack is sufficiently wide to neglect the effect of concrete on the fiber corrosion process. During the exposure of RSFs to dry–wet cycles, most fibers ruptured, before the 90 days of chloride exposure had ended. For the RSFs fully exposed, an irregular reduction of cross section was observed along their length due to localized corrosion and accretion of corrosion products at RSFs’ surface (rough fiber surface) during the drying phase (mass loss of 0.31 mg/mm). For RSFs with an exposed length of 0.5 mm and 1.0 mm, the local corrosion was accelerated since the mass loss was higher (2.94 mg/mm and 4.65 mg/mm, respectively) and caused the rupture of all fibers for a shorter exposure time (47 days and 52 days, respectively). Significant dispersion of the results was also observed, which indicates an irregular character of RSFs to corrosion susceptibility when the fibers are partially exposed to aggressive environmental conditions. The chloride exposure conditions adopted are also supported by the study of Mangat and Gurusamy [34], who found that most of the ingress of chlorides took place during the first three months, and at crack width above 0.5 mm the effect is significant.

The RPT-3ps were divided into two series, considering the distinct target pre-crack width, as summarized in Table 2. For each test series, three RSFRC panels submitted to chloride attack (Cl^−^) and three reference RSFRC panels (REFs) were tested. The reference panels and those submitted to chloride attack were produced from different batches.

The chloride or water immersion was carried out in 1000 L tanks, where the panels were positioned horizontally according to the RPT-3ps test configuration, i.e., supported at the same three points used for the pre-cracking process. After completing the adopted exposure period, the panels were submitted to the final RPT-3ps to assess the influence of chloride attack in the post-cracking behavior of RSFRC. The panels were supported on the same three-point supports, and a central point load was applied at a constant displacement rate of 4 mm/min up to a central displacement of 40 mm.

##### 2.3.2.4. Fiber Distribution/Orientation

For a better understanding of the residual stresses and energy absorption obtained in the post-exposure RPT-3ps, fiber distribution, and orientation parameters were determined by image analysis on plane surfaces of the tested specimens, according to the procedure adopted by Frazão et al. [3], Abrishambaf [35] and Cunha [36]. After performing the post-exposure RPT-3ps, one of the three distinct parts delimitated by the crack surfaces was cut parallel and as close as possible to the crack surface. The applied method consists of recognizing the cross section of each RSF, from the surrounding matrix, by image processing of high-resolution pictures taken from the cut surface of the specimens. After computation of the image analysis results, the following parameters that characterize the fiber distribution and orientation were derived out, namely, (1) the number of fibers per unit area, $N^{f}$, which corresponded to the ratio between the number of counted fibers and the area of the cut surface and (2) fiber orientation factor, η, which intends to simulate the influence of fiber orientation on the response of FRC structural elements and was calculated with two different approaches, as presented in Frazão et al. [3]. The first method was calculated based on the image analysis procedure of the cut surface. In this case, the orientation factor for the fibers intersecting the cut surface, $\eta_{img}$, was determined by using the following Equation (1), where $\theta_{i}$ is the angle between the fiber’s longitudinal axis and the orthogonal to the cut section:

(1) $$\eta_{img}=\frac{1}{N_{}^{f}}\sum_{i=1}^{N^{f}}\cos\theta_{i}$$

In the second method, the fiber orientation factor within a cross section, $\eta_{\exp}$, was obtained from Equation (2) proposed by Soroushian and Lee [37], where $A_{f}$ and $V_{f}$ are the cross-sectional area of a single RSF and the volumetric percentage of fibers added to concrete, respectively.

(2) $$\eta_{\exp}=N_{}^{f}\frac{A_{f}}{V_{f}}$$

## 3. Results and Discussion

### 3.1. Compressive Behavior of RSFRC

**Figure 4:**
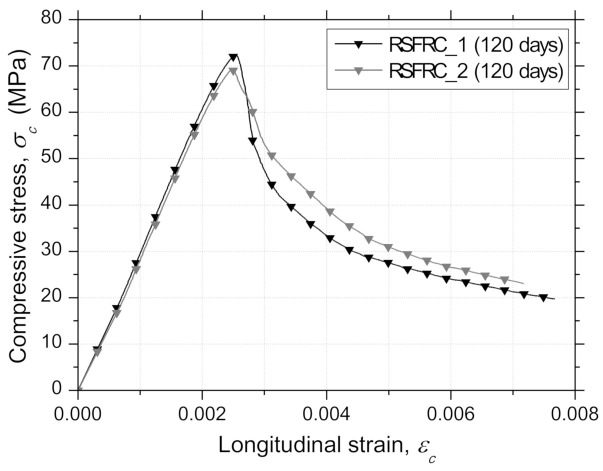
Average compressive stress–longitudinal strain curves obtained in compression tests of RSFRC_1 and RSFRC_2 batches (120 days).

The average $\sigma_{c}-\varepsilon_{c}$ curves determined at the same age of round panel tests (120 days) from a set of four concrete specimens of each RSFRC batch (RSFRC_1 and RSFRC_2) are depicted in Figure 4. Table 3 includes the average values and the corresponding coefficients of variation (CoV) of the hardened density, the elasticity modulus, $E_{cm}$, the compressive strength, $f_{cm}$, the strain at peak load, $\varepsilon_{c1}$, and the energy dissipated under compression, $G_{c}$, calculated as the area under the stress–strain curve, $\sigma_{c}-\varepsilon_{c}$, until an ultimate deformation, $\varepsilon_{u}$, of 0.005, where the residual strength was less than 50% of the corresponding $f_{cm}$. The maximum compressive strength was slightly higher in RSFRC_1 than in RSFRC_2, although this last batch showed a lower residual strength drop after the peak load.

Immediately after the peak load, a high gradient of stress decay was observed in the RSFRC since fibers were not able to sustain the relatively high release of energy in the RSFRC at this stage. The average values of $E_{cm}$ and $f_{cm}$ were also slightly lower for batch RSFRC_2 (Table 3). In general, low CoV values were obtained for all the evaluated parameters in the compression tests, which attests to the adequate homogeneity of produced concrete.

### 3.2. Flexural Behavior of RSFRC

**Figure 5:**
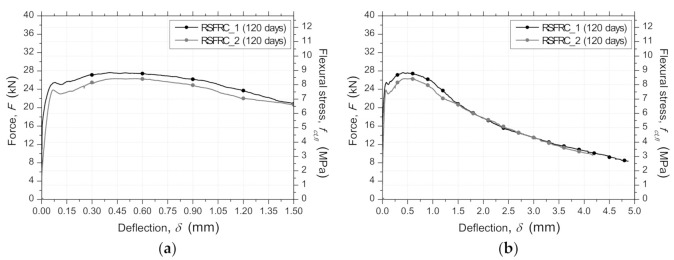
Average force/flexural stress–deflection curves obtained in bending tests of RSFRC_1 and RSFRC_2 batches (120 days). (**a**) Deflection up to 1.5 mm (**b**) Deflection up to 5 mm.

Figure 5 presents the average 120 days force/flexural stress–deflection ($F/f_{ct,fl}-\delta$) relationship, obtained for the notched beams RSFRC_1 and RSFRC_2. Regarding the pre-peak behavior, after the cracking load has been attained, the RSFRC beams presented a small decrease of flexural capacity until a deflection at which the fiber reinforcement mechanisms started being mobilized and controlling the crack opening process (Figure 5).

From the 3PNBBT, the residual flexural tensile strength parameters ($f_{R,j}$) were computed according to *fib* Model Code 2010 recommendations [32]. Based on the load values, $F_{j}$, corresponding to the $CMOD_{j}$ (j = 1 to 4 equal to 0.5 mm, 1.5 mm, 2.5 mm, and 3.5 mm, respectively), the parameters $f_{R,j}$ were determined from the following equation:

(3) $$f_{R,j}=\frac{3\;F_{j}\;L}{{2\;b\;h}_{\mathrm{sp}}^{2}}$$

Table 4 shows the average values and the corresponding coefficients of variation (CoV) of the flexural tensile strength parameters, $f_{R,1}$, $f_{R,2}$, $f_{R,3}$ and $f_{R,4}$, considering the two concrete batches. From the data presented in Table 4, in general, no significant differences were observed between the residual strengths of RSFRC_1 and RSFRC_2. On average, the peak load was about 5% higher in RSFRC_2 than in RSFRC_1, corroborating the slightly higher mechanical strength of this batch.

### 3.3. Round Panel Tests under Chloride Attack

#### 3.3.1. Chloride Penetration into RSFRC Panels

**Figure 6:**
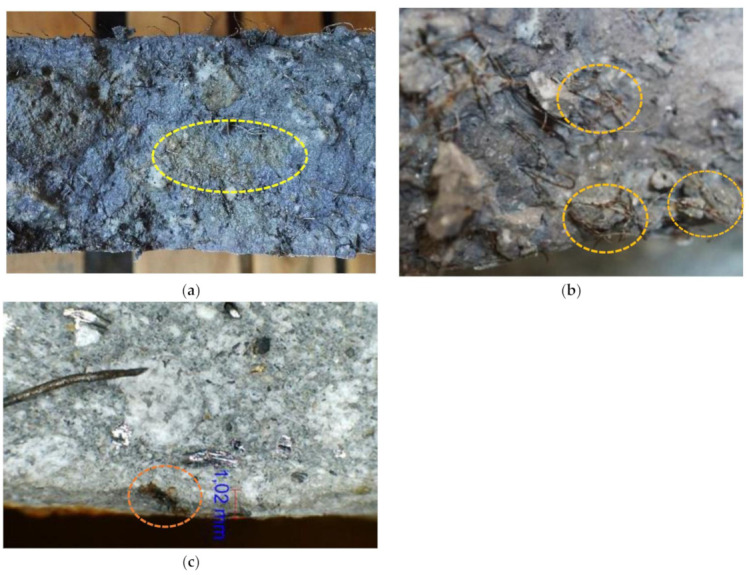
(**a**) Inspection of recycled steel fibers’ (RSFs) corrosion at the crack surface; (**b**) chloride penetration depth at the cracked surface; and (**c**) corrosion signs on RSFs located on an orthogonal cut plane to a crack surface (uncracked region), after the exposure period of 90 days of dry–wet cycles.

After 90 days of dry–wet cycles in chloride solution, a significant increase of corrosion spots occurred on the surface of pre-cracked RSFRC panels. At the end of RPT-3ps, the cracked surfaces of the RSFRC panels submitted to chloride exposure were visually observed. To assess the chloride penetration depth, the crack surfaces of these panels were sprayed with silver nitrate solution. The cracked surfaces were almost completely penetrated by chlorides during the immersion period, only a few irregular areas inside the panels showed no signs of chloride penetration (Figure 6a).

As shown in Figure 6b, some RSFs at cracked surfaces presented corrosion products, mainly near the bottom of the exposed face of the panels, where the crack width was higher and closer to the chloride solution. It seems that the RSFs’ corrosion occurred mainly at the fiber length crossing the crack width (in direct contact with the chloride solution) (Figure 6b). However, few corrosion spots were detected by microscopic inspection on RSFs up to 1–3 mm deep, located on a cut surface orthogonal to a crack, as observed in Figure 6c.

#### 3.3.2. Evaluation of Crack Width Measurements

**Figure 7:**
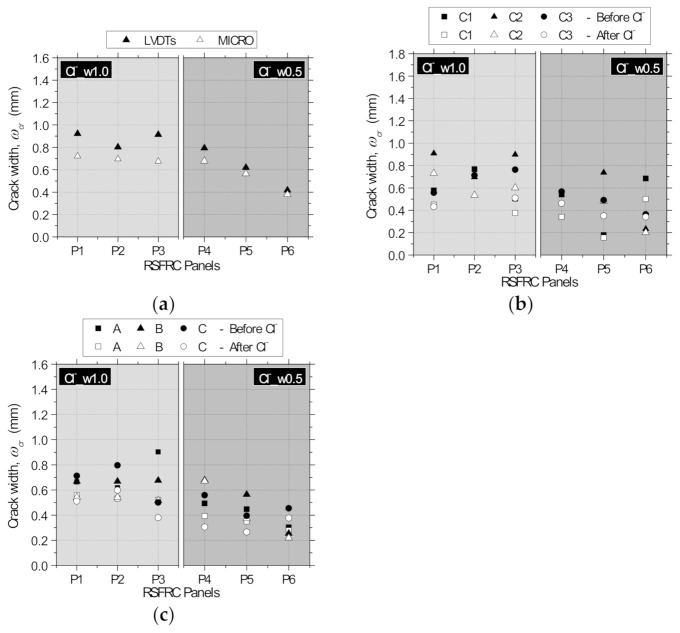
Crack width measurements performed with (**a**) the LVDTs and the microscope; (**b**) the microscope at each crack C1, C2, and C3 (Figure 3d); and (**c**) the microscope at each position A, B, and C (Figure 3d).

As mentioned in Section 2.3.2.1, the crack widths were also measured with a USB microscope before and after submitting the panels to immersion. The measurements were performed at three points of each crack in the bottom surface of the panel, as schematically represented in Figure 3d (points A, B, and C). In some of these positions, it was not possible to measure the crack width due to the difficulty in capturing a sharp image with well-defined crack boundaries. For this reason, only reliable results of crack width measured with the microscope are presented. Since the observations in terms of crack width measurement were similar between the series subjected to different environmental exposure conditions (Cl^−^and REF), only the results obtained in panels of Series Cl^−^_w0.5 and Cl^−^_w1.0 are graphically presented. Figure 7a shows the comparison between the crack width measurements performed with the LVDTs and with the microscope at position B (Figure 3d) after pre-cracking and before immersion. The results represent the average of the three radial cracks of the panels. According to the results obtained in all test series, the crack widths measured with the microscope were slightly lower than that measured with the LVDTs, with an average difference of 0.14 mm for the panels with the higher pre-crack width level (panels P1, P2, and P3) and 0.11 mm for the panels with the smaller pre-crack width level (panels P4, P5, and P6). The larger crack width values measured by the LVDTs is caused by the elastic deformation included in the registered measures and due to the kinematic mechanism mentioned by Spasojević [33], which owed to the geometry of the measurement device, l m, in which rotation of the measurement base, *θ*, caused higher experimental captured deformations, *l_m_* + ∆*l_m,device_*, than the real element tensile deformations, *l_m_* + ∆*l_m_* (Figure 2c).

Figure 7b represents the comparison between the crack width measurements performed with the microscope for each crack, C1, C2, and C3 (average of the three measurements at positions A, B, and C; Figure 3d), before and after continuous immersion/dry-wet cycles, i.e., before introducing the panels into the tanks and after removing them from the tanks and placing in the test setup for the final RPT-3ps. According to the results of Figure 7b, a slight reduction of the crack width occurred in the three cracks of the panels after the chloride exposure (on average, 0.10 mm for the panels with the higher pre-crack width level and 0.07 mm for the panels with the smaller pre-crack width level). High dispersion of the results was observed between the measurements at the three different cracks (CoV ranging from 3% to 60%), probably due to the irregular character of RSFs’ geometry crossing the cracks.

Figure 7c shows the comparison between the crack width measurements performed with the microscope at each position, A, B, and C (average of the three measurements at cracks C1, C2, and C3; Figure 3b), before and after continuous immersion/dry–wet cycles. A slight reduction of the crack width was observed at the three crack positions A, B, and C of the panels after performing the environmental exposure (on average, 0.10 mm for the panels with the higher pre-crack width level, and 0.06 mm for the panels with the smaller pre-crack width level). High dispersion of the results was also observed between the crack measurements at the three different positions (CoV ranging from 2% to 42%). Since the difference between the average crack width measured at the center of the panel (position A) and at the edge of the panel (position C) was within the test variability and did not follow a clear trend, the crack width along the crack development length was assumed constant in the theoretical approach for deriving the stress vs. crack width by inverse analysis RPT-3ps (Section 4).

#### 3.3.3. Force–Central Deflection Relationship

**Figure 8:**
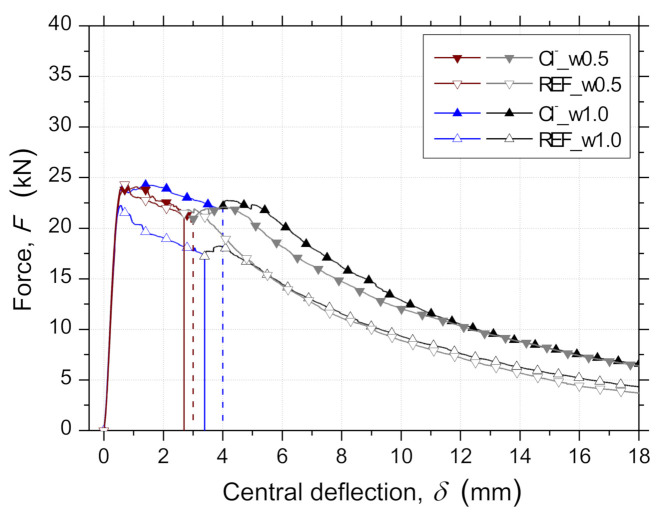
The average force–central deflection (F−δ) relationship for panels from RPT-3ps during the pre-cracking process and after the environmental exposure period of 90 days.

Figure 8 presents the average force–central deflection responses, F−δ, registered in the RSFRC panels, during the pre-cracking process (initial load/unload cycle) and after the environmental exposure period of 90 days of dry–wet cycles/immersion. The average pre-crack widths, $\omega_{cr}$ (after unloading), based on microscope measurements, are indicated in Table 5 and were close to the target crack widths of 0.5 mm and 1.0 mm.

The pre- and post-cracking load-carrying capacity observed for the panels submitted to chloride attack for 90 days of dry–wet cycles (RSFRC_1 batch) was higher than that of the corresponding pre-cracked reference panels (Figure 8). This corroborates the 3PNBBT results (Section 3.2) and contributes to the higher post-cracking load-carrying capacity of pre-cracked panels subjected to chloride attack.

**Figure 9:**
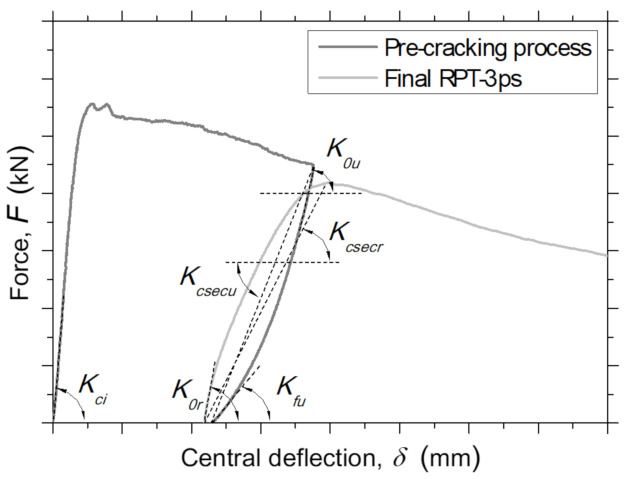
Stiffness parameters determined for each pre-cracked panel using the obtained experimental data.

For the F−δ relationship obtained in each pre-cracked panel, the parameters of stiffness represented in Figure 9 were determined, namely, the initial stiffness, $K_{ci}$, initial unloading tangent stiffness, $K_{0u}$, final unloading tangent stiffness, $K_{fu}$, initial reloading tangent stiffness, $K_{0r}$, unloading secant stiffness, $K_{\mathrm{csecu}}$, and reloading secant stiffness, $K_{\mathrm{csecr}}$. The results obtained of the normalized stiffness parameters are presented in Table 5.

Comparing the pre-cracked chloride-immersed (Cl^−^) panels with the corresponding reference (REF) pre-cracked panels, no significant differences were observed between the stiffness parameters, which suggests that the corrosion of RSFs had a negligible effect on the post-cracking behavior of cracked RSFRC up to a crack width of 1 mm, under the adopted exposure periods. The small differences observed between the stiffness parameters obtained in the pre-cracked panels may be justified by the differences between the pre-crack width levels, fiber distribution, and test variability. In addition to this aspect and the RSFRC characteristics, the F−δ relationship is also affected by the panel thickness. This is analyzed further in the next sections.

#### 3.3.4. Energy Absorption–Central Deflection Relationships

**Figure 10:**
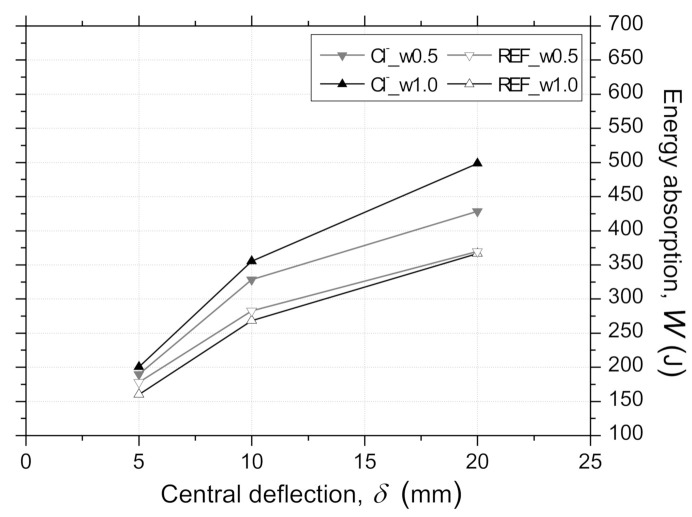
Average energy absorption–central deflection (W − δ) relationships for pre-cracked panels from RPT-3ps after the exposure period of 90 days.

The average values of energy absorbed by the RSFRC panels, W, up to a central deflection, δ, of 5 mm, 10 mm, and 20 mm are presented in Figure 10. These W values were corrected considering the panel thickness by using Equation (4) recommended by ASTM C1550-08 [27], where W is the corrected energy absorption (J), $W^{\prime }$ is the measured energy absorption (J), $t_{0}$ is the nominal thickness of 75 mm, t is the measured panel thickness (mm), $d_{0}$ is the nominal diameter of 800 mm, d is the measured panel diameter (mm), and δ is the specified central deflection at which the energy absorption is evaluated (mm).

(4) $$W=W^{\prime }{\left(\frac{t_{0}}{t}\right)}^{\beta }\left(\frac{d_{0}}{d}\right)\;\mathrm{with}\;\beta \;=\;2.0\;-\left(\delta \;-\;0.5\right)/80$$

According to Figure 10, the panels submitted to 90 days of dry–wet cycles in chloride solution showed higher absorbed energy than the corresponding reference ones, and this difference has increased with the pre-crack width. The obtained results may be affected by the fiber distribution and orientation at crack surfaces. This is further discussed in Section 3.3.6.

#### 3.3.5. Force–Crack Width Relationships

**Figure 11:**
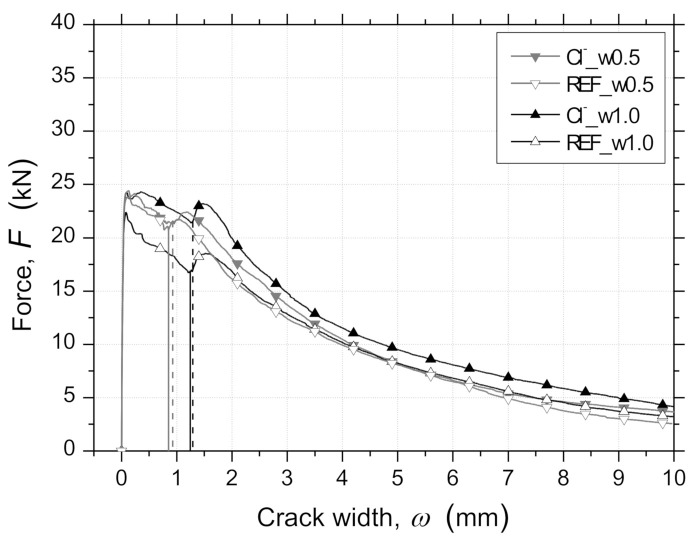
Average force–crack width (F−ω) relationships for panels from RPT-3ps after the exposure period of 90 days.

Figure 11 represents the average force–crack width responses, F−ω, registered on the RSFRC panels. The crack width, ω, corresponds to the mean value measured by the three LVDTs used for measuring the width of the three developed cracks (Figure 3c). Until the occurrence of the first crack in the measuring stroke of the LVDT, the displacement recorded was an elastic deformation of the RSFRC panels, but this is a very smaller parcel, even when compared to the smallest pre-crack width values adopted.

No significant differences were observed between the average F−ω relationships of pre-cracked panels with different $\omega_{cr}$ submitted to 90 days of environmental exposure. This is indicative of a negligible effect of the chloride attack, and, consequently, of the RSFs’ corrosion products on the average progression of crack widths during the RPT-3ps for the predefined conditions of chloride exposure and pre-crack width level.

#### 3.3.6. Fiber Distribution/Orientation Profile

Table 6 includes the fiber distribution and orientation parameters obtained from pre-cracked round panels. For a better analysis of the values of $N^{f}$, the corresponding energy absorption registered in the analyzed panels, in addition to an estimate of the percentage of fibers that failed by rupture (assuming that the fibers with visible length counted at the crack surface had failed by pull-out), are also depicted in Table 6.

The obtained values of $\eta_{img}$ and $\eta_{exp}$ did not significantly vary between panels, which means that no significant differences in fiber orientation occurred. The obtained values of $\eta_{img}$ were higher than the correspondent $\eta_{exp}$, which may be justified by the higher difficulty in detecting the $N^{f}$ according to this approach. No significant differences were observed between the orientation factors of the different test series, which means that the fiber orientation at crack surfaces had a negligible effect on the results of RPT-3ps, excluding the hypothesis put forth in Section 3.3.3, with the claim that the differences between the stiffness parameters obtained in the pre-cracked panels were due to the differences of fiber distribution in the panels.

In addition, after 90 days of environmental exposure, similar values of $N^{f}$ and percentage of fibers that failed by rupture were obtained for chloride-attacked and reference panels. This would suggest that the differences between the corresponding energy absorption (Table 6) can be also attributed to the differences between the $N^{f}$ and percentage of failed fibers by rupture at the three cracks in the analyzed panels since only one crack surface of each analyzed panel was considered to study the fiber density.

Due to the strong bond between recycled fibers and matrix, a high percentage of RSFs failed by rupture, as indicated in Table 6, which is indicative of a high bond stiffness between the fibers and the concrete matrix. The percentage of fibers that failed by rupture was similar for Cl^−^ and REF specimens, which is also indicative of a negligible effect of chloride attack in this respect.

In conclusion, despite the high crack widths and the severe exposure conditions considered in this study, after three months of chloride attack, the corrosion was limited to the crack region and without significantly reducing the fiber section. Therefore, RSFRC showed to be able to effectively retain the crack propagation and to delay the subsequent fiber chloride corrosion during a reasonable period after the first cracking. This is especially attractive in controlling the surface cracking of structural elements under chloride attack, conserving the RSFRC properties during current long-term deferred actions.

## 4. Numerical Simulations

Numerical simulations were performed to obtain the post-cracking constitutive laws of the developed RSFRC, derived from the inverse analysis by fitting the experimental results obtained in 3PNBBT and RPT-3ps, which knowledge may contribute to future design guidelines and design tools for RSFRC structures under aggressive chloride exposure conditions. The fracture parameters define the σ−ω constitutive law that governs the fracture propagation of the RSFRC when using a FEM-based model [38], a cross-sectional approach [39], or any formulation capable to simulate the contribution of the post-cracking tensile capacity of a cement-based material for the verifications at the serviceability and at ultimate limit state conditions [20].

### 4.1. Evaluation of the Mode I Fracture Parameters from Inverse Analysis of 3PNBBT

The experimental force–crack mouth opening displacement (F−CMOD) curves obtained with RSFRC_1 and RSFRC_2 notched beams at 120 days were simulated using a numerical model developed in previous research [36,40], implemented in the FEMIX, a software based on the finite element method [41]. Due to the geometry, support, and loading conditions used in the 3PNBBT, a plane stress state was assumed in the beam. For the numerical simulation of the crack initiation and propagation, 2D line interface elements of six nodes located on the symmetry axis of the specimen were used. The remaining part of the specimen was modeled with a mesh of eight-node serendipity plane stress finite elements, assuming a linear elastic behavior for the material.

**Figure 12:**
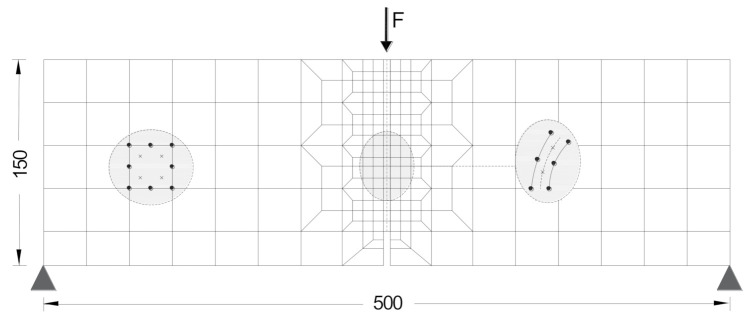
Finite element mesh used in the simulation of the 3PNBBT [36].

The Gauss–Legendre integration scheme with 2 × 2 integration points (IP) was used in all elements, with exception of the interface finite elements at the symmetry axis of the specimen, where a 1 × 2 Gauss–Lobato IP were used in order to assure the crack progress along the symmetry axis. Figure 12 depicts the mesh used in the numerical simulations. The values of the material properties used in the inverse analysis are indicated in Table 7.

**Figure 13:**
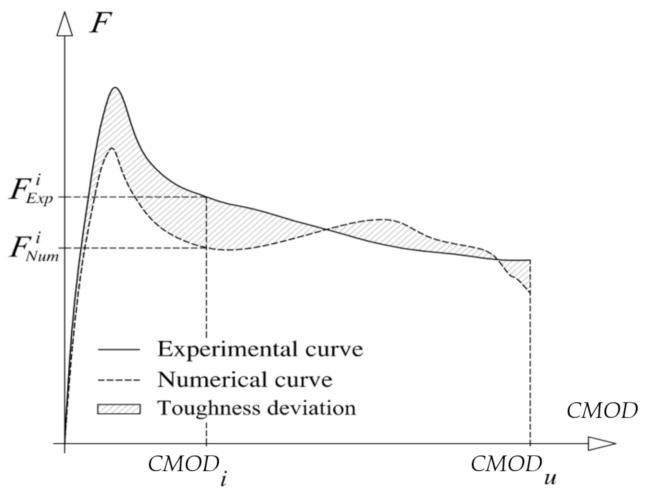
Schematic representation of the force–crack mouth opening displacement (F−CMOD) registered experimentally and obtained numerically by the inverse analysis.

The fracture mode I propagation of RSFRC was simulated by a trilinear tensile–softening (σ−ω) diagram, whose parameters that define the shape rilof the diagram, namely, the mode I fracture energy, $G_{f}$, and the values of crack opening, $\omega_{i}$, and corresponding tensile stress, $\sigma_{i}$, were obtained by performing inverse analysis by fitting the average F−CMOD relationship obtained experimentally in the performed 3PNBBT with a target tolerance. The objective of the inverse analysis is to evaluate the fracture mode I parameters, by attending two convergence criteria based on the approximation of the F−CMOD registered experimentally and determined numerically by the FEM simulations. For this purpose, FEM analyses are automatically executed for the assumed interval of values (minimum and maximum) of the parameters that define the σ−ω, namely, $f_{ct}\in \left[f_{ct,\min}-f_{ct,\max}\right]$, $\omega_{1}\in \left[\omega_{1,\min}-\omega_{1,\max}\right]$, $\sigma_{1}\in \left[\sigma_{1,\min}-\sigma_{1,\max}\right]$, $\omega_{2}\in \left[\omega_{2,\min}-\omega_{2,\max}\right]$, $\sigma_{2}\in \left[\sigma_{2,\min}-\sigma_{2,\max}\right]$ and $\omega_{u}\in \left[\omega_{u,\min}-\omega_{u,\max}\right]$, at selected increments, $\Delta f_{ct}$, $\Delta \omega_{1}$, $\Delta \sigma_{1}$, $\Delta \omega_{2}$, $\Delta \sigma_{2}$, $\Delta \omega_{u}$, respectively. For each set of combination of these parameters, the deviation between the F−CMOD registered experimentally and determined numerically is calculated (Figure 13) in terms of force values and area behind the F−CMOD as follows:

(5) $$err_{F}=\frac{\left|F_{Exp}^{k}\;-\;F_{Num}^{k}\right|}{F_{Exp}^{k}}$$

(6) $$err_{T}=\frac{\left|A_{Exp}^{{\left(F-CMOD\right)}_{k}}\;-\;A_{Num}^{{\left(F-CMOD\right)}_{k}}\right|}{A_{Exp}^{{\left(F-CMOD\right)}_{k}}}$$

where $A_{Exp}^{{\left(F-CMOD\right)}_{k}}$ and $A_{Num}^{{\left(F-CMOD\right)}_{k}}$ are the area beneath, respectively, the experimental and numerical F−CMOD curves up to $CMOD^{k}$ that can be obtained from the following equations:

(7) $$A_{Exp}^{{\left(F-CMOD\right)}_{k}}=\sum_{i=2}^{k}0.5\left(F_{Exp}^{i}+F_{Exp}^{i-1}\right)\left(CMOD_{i}-CMOD_{i-1}\right)$$

(8) $$A_{Num}^{{\left(F-CMOD\right)}_{k}}=\sum_{i=2}^{k}0.5\left(F_{Num}^{i}+F_{Num}^{i-1}\right)\left(CMOD_{i}-CMOD_{i-1}\right)$$

The set of parameters’ values that are conducted to the smallest $err_{F}$ and $err_{T}$ are those considered defining the σ−ω of the RSFRC.

**Figure 14:**
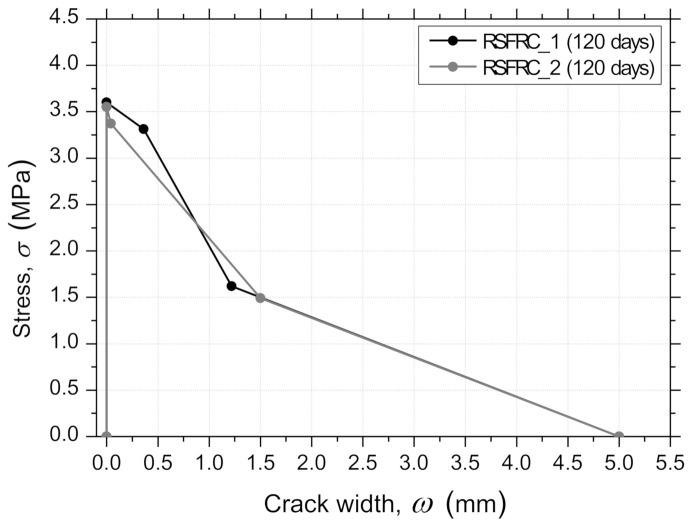
Trilinear tensile–softening (σ−ω) relationships obtained by inverse analysis from 3PNBBT.

The obtained parameters defining the σ−ω relationship, and the equivalent fitting error, e, are indicated in Table 8, where $f_{ct}$ is the tensile strength, $\sigma_{1}$ and $\sigma_{2}$ are, respectively, the stress at the first and second post-peak point of the crack opening, $\omega_{1}$ and $\omega_{2}$, and $\omega_{u}$ is the ultimate post-peak point of the crack opening. The graphical representation of these σ−ω laws is presented in Figure 14. For the same crack width level, no significant differences in the parameters were observed (Figure 14).

### 4.2. Evaluation of the Mode I Fracture Parameters from Inverse Analysis of RPT-3ps

**Figure 15:**
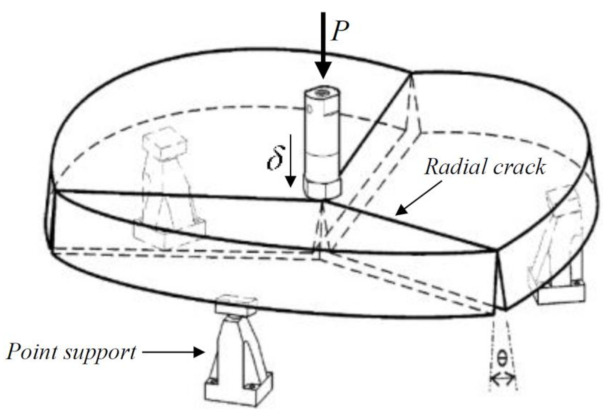
RPT-3ps setup with three-point support and normal failure mode (*P* and *δ* represent, respectively, the central load and deflection) [42].

From the inverse analysis, the post-cracking constitutive laws of the RSFRC representative of the RPT-3ps were determined by fitting the experimental curves of the average F−δ relationships obtained in each test series of RPT-3ps. This strategy allowed to evaluate the influence on the σ−ω relationship of the chloride exposure conditions adopted for the pre-cracked RSFRC round panels. This numerical simulation considers the constitutive laws of RSFRC in tension and in compression to determine the moment–rotation relationship, the loading and support conditions of RPT-3ps (Figure 15), kinematic assumptions, and the principle of virtual work in order to derive the σ−ω law of RSFRC by fitting as much as possible the force–deflection registered in the round panel tests [8,22].

**Figure 16:**
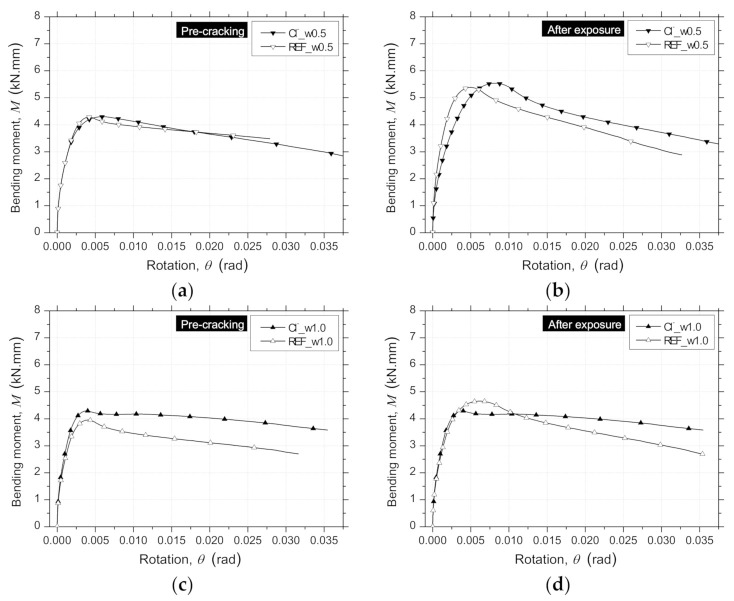
Moment–rotation (*M* − *θ*) relationships obtained by inverse analysis from RPT-3ps of RSFRC panels with the target *ω_cr_* of 0.5 mm (**a**) during the pre-cracking stage and (**b**) after environmental exposure; and the target *ω_cr_* of 1.0 mm (**c**) during the pre-cracking stage and (**d**) after environmental exposure.

By using a cross-section layer model available in DOCROS computer program (DOCROS—“Design Of CROss Section”, Guimarães, Portugal [43]), the moment–rotation relationship for the round panel cross section was evaluated, as presented in Figure 16. This layered model in DOCROS allows considering sections of irregular shape and size, composed of different types of materials subjected to an axial force and variable curvature or crack width [43]. The compression and tensile behavior of each material can be simulated by several types of constitutive laws. To obtain the moment–rotation of the RPT-3ps, a round panel’s cross section of 1000 mm wide and a height corresponding to the average thickness of the tested panels was considered, having this height been discretized in 60 layers of equal thickness.

In the numerical model used, proposed by Salehian et al. [8,22], it is assumed that in RPT-3ps, just after the peak load, three dominant cracks propagate in the panel, subdividing it into three rigid plates (Figure 15 [42]), whose elastic deformation is recovered in the structural softening stage when cracks are opening gradually. The elastic deformation that occurred in the pre-cracking stage of RSFRC panels (the deformation registered up to the central deflection of 0.17 mm, on average) was neglected because it was much lower than the deflection imposed to implement the target crack width (3.15 mm on average). Therefore, the vertical deformation of the panel’s center, *δ*, was attributed to the rigid rotation of the plates in turn of their connecting dominant cracks. For the sake of simplicity, it is assumed that the cracks are straight and radiate from the panel’s center (Figure 15) [8,22]. The model considers work equilibrium conditions, the tensile properties of RSFRC, and uses the above-mentioned moment–rotation approach. Further details on this numerical model can be found in Salehian et al. [8,22].

**Figure 17:**
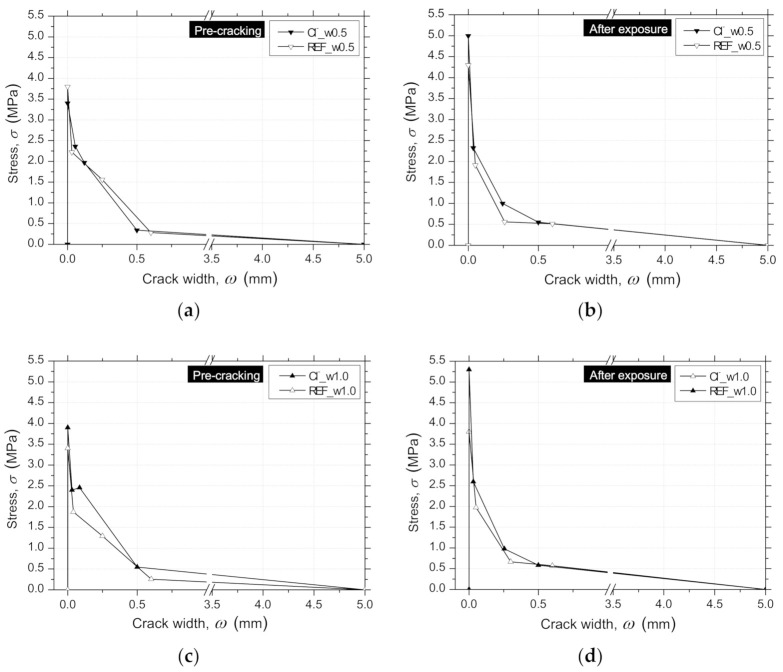
Stress–crack width (σ−ω) relationships obtained by inverse analysis from RPT-3ps of RSFRC panels with the target $\omega_{cr}$ of 0.5 mm (**a**) during the pre-cracking stage and (**b**) after environmental exposure; and the target $\omega_{cr}$ of 1.0 mm (**c**) during the pre-cracking stage and (**d**) after environmental exposure.

A quadrilinear tensile–softening (*σ* − *ω*) diagram was used to simulate the fracture mode I propagation of pre-cracked RSFRC, whose parameters were obtained by performing inverse analysis with the average *F* − *δ* relationships obtained for the pre-cracking stage and after the environmental exposure of the pre-cracked panels. The obtained parameters that define the shape of the diagram and the equivalent fitting error, *e*, are indicated in Table 9. The graphical representation of these *σ* − *ω* diagrams is presented in Figure 17a–d.

During the pre-cracking stage of the panels (Figure 17a,c), the determined parameters corroborate with the flexural tensile strength parameters obtained in the 3PNBBT of the corresponding RSFRC beams (Table 4 and Figure 5).

After the exposure period of 90 days, the differences in the fracture energy obtained during the pre-cracking stage (Figure 17a,c) and after the environmental exposure of pre-cracked panels (Figure 17b,d) were mainly explained by the difference in concrete age. This suggests that the action of RSFs’ corrosion was negligible in the post-cracking behavior of cracked RSFRC submitted to these chloride exposure conditions. According to the present study, a longer exposure period for chloride environment (>3 months) should be adopted for a more comprehensive assessment of the long-term effects of chloride attack in cracked RSFRC.

According to the results presented in Frazão et al. [4], concerning the RSFs’ mass loss by non-induced corrosion after seven days of immersion (3.54%) and the values of corrosion potential (*E_(i=0)_* = −631.1 ± 2.2 mV vs SCE-Saturated Calomel Electrode) and corrosion rate (3.715 ± 0.909 mpy) obtained from the linear polarization curves performed on RSFs after seven days of immersion, a high risk of RSFs’ corrosion was evidenced compared to industrial steel fibers. In this sense, a significant reduction in the cross section of the fibers crossing the cracks, in addition to a consequent reduction of the post-cracking behavior of RSFRC, would be expected. However, in the pre-cracked RSFRC panels, the concrete pore solution environment, and the variability of the crack width along its V-shape probably justified the lower effect of corrosive action in the post-cracking behavior of RSFRC found in this research study.

**Figure 18:**
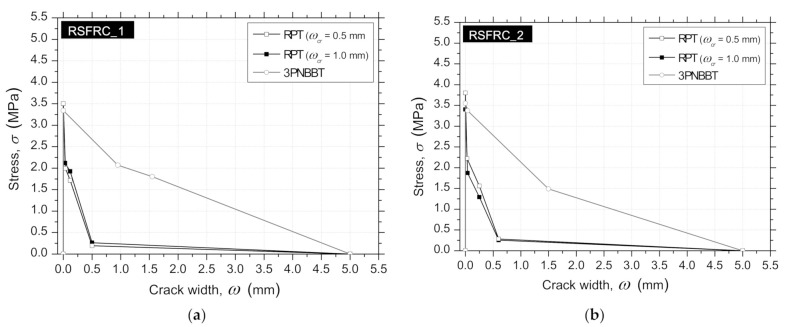
Stress–crack width (σ−ω) relationships obtained by inverse analysis from RPT-3ps and 3PNBBT for: (**a**) RSFRC_1 batch (**b**) RSFRC_2 batch.

Comparing the σ−ω relationship of RSFRC obtained by inverse analysis from RPT-3ps and 3PNBBT presented in Figure 18 and Table 8 and Table 9, it is verified that the constitutive laws obtained from 3PNBBT (prismatic specimens with a localized crack) overestimated the post-cracking behavior of RSFRC compared to the constitutive laws obtained from RPT-3ps, which are more representative of the fiber reinforcement mechanisms developed in thin elements (wall elements, panels, slabs, shotcrete linings).

## 5. Conclusions

The present research involves both experimental and numerical research regarding the post-cracking behavior of cracked RSFRC under chloride attack. The main conclusions based on the experimental and numerical results are as follows:

1. After 90 days of chloride attack, the cracked surfaces of pre-cracked RSFRC panels with crack widths up to 1 mm were completely penetrated by chlorides during the immersion period, and corrosion products were visible in the RSFs located in the cracked surfaces;
2. Significant differences may occur in the progress of the three crack widths in round panels during RPT-3ps due to fiber distribution of RSFs at crack surfaces, with an inherent influence on the energy absorption of RSFRC panels;
3. The stiffness parameters obtained in RSFRC panels indicate that the adopted corrosion induction conditions for RSFs had a negligible effect on the post-cracking behavior of cracked RSFRC up to a crack width of 1 mm;
4. The RPT-3ps revealed small differences between the post-cracking behavior of pre-cracked panels submitted to 90 days of chloride attack and the corresponding pre-cracked reference panels;
5. A high percentage of RSFs failed by rupture in all test series of RPT-3ps, which is indicative of a negligible effect of chloride attack;
6. No significant differences were detected in terms of the fiber orientation factor, between reference panels and panels submitted to chloride attack;
7. Comparing the σ−ω relationships of the RSFRC representative of the RPT-3ps, obtained by inverse analysis procedure for pre-cracking stage and after environmental exposure, the chloride attack for 90 days of dry–wet chloride cycles had a negligible effect on the post-cracking behavior of pre-cracked RSFRC panels with crack widths up to 1 mm;
8. The constitutive laws of the RSFRC representative of the 3PNBBT overestimated the post-cracking behavior of RSFRC comparing with the constitutive laws of the RSFRC representative of the RPT-3ps.

## Figures and Tables

**Table 1:** Mix proportions for 1 m^3^ of recycled steel fiber reinforced concrete (RSFRC).

C (kg)	FA (kg)	FS (kg)	CS (kg)	CG (kg)	W (L)	SP (L)	Cf (kg)	W/C *
400	200	148	735	597	173	7.2	75.8	0.43

* Water-cement ratio.

**Table 2:** The experimental program of RPT-3ps.

Test Series	RSFRC Batch	$\omega_{cr}$ (mm)	Exposure Conditions of Panels before RPT-3ps
Cl^−^_w0.5	RSFRC_1	0.5	90 days of dry-wet cycles in 3.5 wt% NaCl solution
Cl^−^_w1.0		1.0	
REF_w0.5	RSFRC_2	0.5	90 days of tap water immersion
REF_w1.0		1.0	

**Table 3:** Average results (and coefficients of variation (CoV) of compression tests.

RSFRC Batch	Density (kg/m^3^)	$E_{\mathrm{cm}}\;(\mathrm{GPa})$	$f_{cm}\;(\mathrm{MPa})$	$\varepsilon_{c1}$	$G_{c}\;(\mathrm{MPa})$
RSFRC_1	2335.68 (1.04)	34.68 (8.22)	72.90 (2.41)	0.0025 (2.89)	0.19 (10.53)
RSFRC_2	2330.69 (0.61)	32.74 (4.98)	68.96 (3.37)	0.0025 (4.65)	0.20 (5.83)

**Table 4:** Average results (and CoV) of 3PNBBT.

RSFRC Batch	*f_R_*_,1_ (MPa)	*f_R_*_,2_ (MPa)	*f_R_*_,3_ (MPa)	*f_R_*_,4_ (MPa)
RSFRC_1	8.86 (10.89)	7.57 (13.62)	5.71 (16.58)	4.60 (14.83)
RSFRC_2	8.43 (19.89)	7.14 (25.73)	6.18 (18.54)	4.66 (23.93)

**Table 5:** Average results (and CoV) of normalized stiffness parameters.

Test Series	$\omega_{cr}$ (mm)	$K_{\mathrm{ci}}$ (kN/mm)	$K_{0u}{/K}_{\mathrm{ci}}$	$K_{\mathrm{fu}}{/K}_{\mathrm{ci}}$	$K_{0r}{/K}_{\mathrm{ci}}$	$K_{\mathrm{csecu}}{/K}_{\mathrm{ci}}$	$K_{\mathrm{csecr}}{/K}_{\mathrm{ci}}$
Cl^−^_w0.5	0.59 (13.83)	65.67 (7.98)	0.46 (11.25)	0.09 (0.86)	0.44 (17.79)	0.23 (7.66)	0.17 (12.67)
REF_w0.5	0.58 (5.85)	71.60 (15.65)	0.46 (6.37)	0.10 (23.65)	0.53 (19.96)	0.23 (9.43)	0.22 (8.93)
Cl^−^_w1.0	0.85 (6.94)	62.27 (6.24)	0.42 (13.55)	0.08 (12.95)	0.49 (24.78)	0.19 (16.82)	0.17 (10.80)
REF_w1.0	0.89 (4.39)	68.77 (18.52)	0.39 (12.95)	0.07 (7.93)	0.38 (8.46)	0.19 (14.10)	0.15 (19.64)

**Table 6:** Fiber distribution and orientation parameters for the analyzed crack surface after RPT-3ps.

Test Series	Panel Thickness (mm)	$W_{5}$ (J)	$W_{10}$ (J)	$W_{20}$ (J)	$W_{40}$ (J)	$N^{f}$ (Fibers/cm^2^)	$\eta_{img}$	$\eta_{\exp}$
Cl^−^_w0.5	63.17	204.23	368.23	533.69	627.50	7.35 (88% *)	0.628	0.361
REF_w0.5	64.92	187.52	306.35	427.24	509.51	7.68 (80% *)	0.617	0.377
Cl^−^_w1.0	64.13	200.94	359.01	507.61	598.53	8.42 (73% *)	0.614	0.413
REF_w1.0	65.45	171.95	291.42	407.27	472.62	8.65 (78% *)	0.595	0.424
Average CoV (%)	64.42 1.54	191.16 7.69	331.25 11.49	468.95 13.05	552.04 13.22	8.03 (80% *) 7.62	0.614 2.24	0.394 7.53

* Percentage of fibers failed by tensile rupture.

**Table 7:** RSFRC properties used in the numerical simulation of the 3PNBBT.

Density	Poisson’s Ratio	Young’s Modulus	Tensile Strength	Fracture Mode I Parameters
*ρ* = 2.34 × 10^−5^ N/mm^3^	$\nu_{c}=0.20$	$E_{c}=28,510\;\mathrm{MPa}$	Inverse analysis	Inverse analysis

**Table 8:** Parameters of the *σ* − *ω* relationship obtained by inverse analysis of the 3PNBBT.

Concrete Mixtures	*f_ct_* (MPa)	*σ*_1_ (MPa)	*σ*_2_ (MPa)	*ω*_1_ (mm)	*ω*_2_ (mm)	*ω_u_* (mm)	*G_f_* (N/mm)	*e* (%)
RSFRC_1	3.60	3.31	1.62	0.36	1.22	5.00	6.43	1.15
RSFRC_2	3.55	3.37	1.49	0.04	1.50	5.00	6.30	0.71

**Table 9:** Parameters of the *σ* − *ω* relationship obtained by inverse analysis of the RPT-3ps.

Test Stage	Series	*f_ct_* (MPa)	*σ*_1_ (MPa)	*σ*_2_ (MPa)	*σ*_3_ (MPa)	*ω*_1_ (mm)	*ω*_2_ (mm)	*ω*_3_ (mm)	*ω_u_* (mm)	*G_f_* (N/mm)	*e* (%)
Pre-cracking stage *ω_cr_* = 0.5 mm	Cl^−^	3.40	2.36	1.97	0.34	0.06	0.12	0.50	5.00	1.50	3.88
	REF	3.80	2.22	1.56	0.29	0.03	0.25	0.60	5.00	1.46	3.41
After environmental exposure	Cl^−^	5.00	2.33	1.00	0.55	0.04	0.25	0.50	5.00	1.91	0.23
	REF	4.30	1.91	0.56	0.52	0.05	0.26	0.60	5.00	1.73	0.07
Pre-cracking stage *ω_cr_* = 1.0 mm	Cl^−^	3.90	2.40	2.46	0.55	0.03	0.09	0.50	5.00	2.08	2.78
	REF	3.40	1.87	1.29	0.26	0.04	0.25	0.60	5.00	1.27	3.58
After environmental exposure	Cl^−^	5.30	2.60	0.98	0.58	0.03	0.26	0.50	5.00	2.02	0.38
	REF	3.80	1.98	0.67	0.57	0.05	0.30	0.60	5.00	1.91	0.33

## Data Availability

The data presented in this study are openly available in RepositóriUM (Institutional repository of the University of Minho), at http://hdl.handle.net/1822/66884.
